# Structural and electronic characterisation of π-extended tetrathiafulvalene derivatives as active components in field-effect transistors†
†Electronic supplementary information (ESI) available: Experimental procedures, characterization data, XRD single crystal data, computational details and device fabrication. CCDC 1460868 and 1460869. For ESI and crystallographic data in CIF or other electronic format see DOI: 10.1039/c6ce01200k



**DOI:** 10.1039/c6ce01200k

**Published:** 2016-07-12

**Authors:** Antonio Campos, Neil Oxtoby, Sergi Galindo, Raphael Pfattner, Jaume Veciana, Stefan T. Bromley, Concepció Rovira, Marta Mas-Torrent

**Affiliations:** a Institut de Ciència de Materials de Barcelona (ICMAB-CSIC) , and Networking Research Center on Bioengineering, Biomaterials and Nanomedicine (CIBER-BBN) , Campus Universitari de Bellaterra , Cerdanyola , E-08193 Barcelona , Spain . Email: cun@icmab.es ; Email: mmas@icmab.es ; Fax: +34 935 805 729 ; Tel: +34 935 801 853; b Departament de Ciència de Materials i Física Química & Institut de Química Teòrica i Computacional (IQTCUB) , Universitat de Barcelona , E-08028 Barcelona , Spain; c Institució Catalana de Recerca i Estudis Avançats (ICREA) , E-08010 Barcelona , Spain

## Abstract

The electronic and structural properties of two tetrathiafulvalene derivatives bearing aromatic benzene rings are reported. Thin film transistors of these materials show p-type characteristics with comparable mobility values. It is found that the rigidification of the molecule is beneficial for reducing the reorganisation energy but also has an unfavorable impact on the electronic structure dimensionality.

## Abstract 2

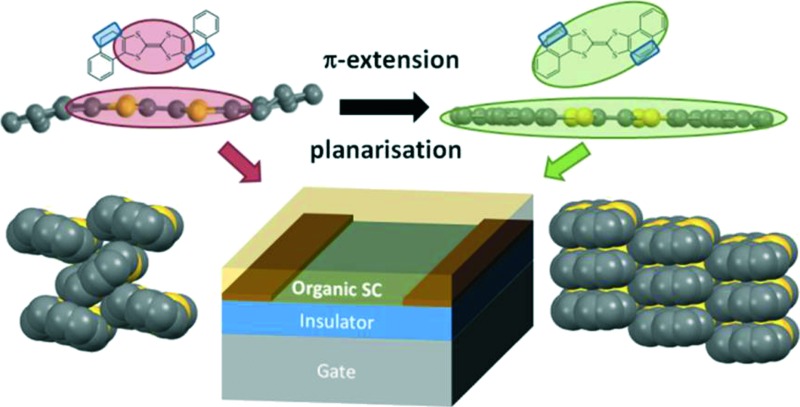
The rigidification of the molecule in π-extended tetrathiafulvalenes is beneficial for reducing the reorganisation energy but has an unfavorable impact on the electronic dimensionality, as observed in the resulting transistors.

Organic semiconductors have attracted a lot of attention in the last two decades because of their high potential as active components in low cost electronics such as RFID tags, sensors or displays.^1^ Further, organic materials offer the possibility that by chemically modifying their molecular cores their electronic and structural characteristics can be tuned.

In particular, the fast development of organic field-effect transistors (OFETs) has resulted in devices exhibiting mobilities equalling or even surpassing that of amorphous silicon.^2,3^ One of the main pillars for progressing in this field is thus the design of novel semiconductors in order to gain further insights into the structural or electronic molecular properties required for achieving high device performance.^4^ It is generally agreed that the transport mechanism in organic semiconductors is dominated by the hopping transport. According to Marcus theory,^5^ the efficiency of hopping conduction is determined by the electronic intermolecular interactions evaluated through the transfer integral (*t*), which needs to be maximised, and the reorganisation energy (*λ*) which has to be minimised. Typically, more planar and rigid molecular systems will give rise to lower *λ* values since the molecular geometry of the charged and neutral molecule will be more similar.^6,7^ On the other hand, the transfer integral is a variable which generally depends on the relative positions and orientations of neighbouring molecules and the shapes of their frontier orbitals.^8^ It is expected though that the extension of the π-conjugation improves the intermolecular electronic interactions and, thus, it is beneficial to charge transport. Therefore, the design of organic semiconductors with fused π-extended systems would, in principle, lead to low *λ* and high *t* values and hence provide a promising route to achieve high mobility OFETs.^9^ However, in order to realise molecular-performance correlation studies, it is imperative to perform systematic studies carrying out controlled molecular modifications in the organic semiconductor backbone. Tetrathiafulvalene (TTF) derivatives have been shown to be excellent candidates for organic semiconductors in OFETs.^10,11^ TTFs are good electron donor molecules that show a high tendency to form ordered stacks or two-dimensional sheets, which are stabilized by both intermolecular π–π and S···S interactions. This family of molecules is generally soluble in conventional organic solvents and can be subjected to a large number of synthetic transformations, a key aspect for the design of customized active materials for potential devices.^12^

In this paper we report an experimental and theoretical study of two TTF derivatives, namely bis(4,5-dihydronaphto[1,2-*d*])tetrathiafulvalene (BDHN-TTF) and bis(naphtho[1,2-*d*])tetrathiafulvalene (BN-TTF) as organic semiconductors in OFETs (Scheme 1). Both molecules mainly differ in the extension of the π-system in the TTF core. It is observed that both materials reveal a p-type semiconductor behaviour. Although the device performance is rationalized in terms of reorganisation energy and transfer integral, it is clearly elucidated that the electronic dimensionality also plays a crucial role here.

**Scheme 1:**
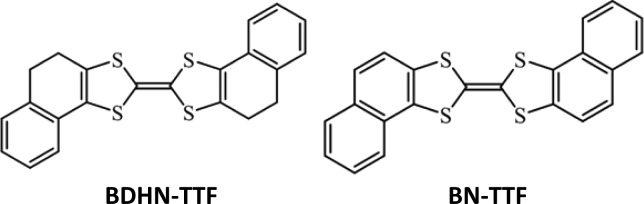


BDHN-TTF was purchased from Aldrich and BN-TTF was synthesized by oxidation of BDHN-TTF with 2,3-dichloro-5,6-dicyano-1,4-benzoquinone (DDQ) in dry toluene under an inert atmosphere refluxing for 20 hours. After recrystallisation in toluene, BN-TTF was obtained in 61% yield (see the ESI†).

The electronic properties of the two TTF derivatives were investigated by cyclic voltammetry (CV) using Ag/AgCl as the reference electrode, a platinum wire as the working and counter electrode and a 0.1 M solution of tetrabutylammonium hexafluorophosphate in CH_2_Cl_2_ as the electrolyte. In the voltammogram of BDHN-TTF and BN-TTF, two reversible oxidation peaks were observed, attributed to the sequential oxidation of the TTF to the radical cation species and the dication (Table 1). It is noticed that the oxidation of BN-TTF takes place at a higher voltage than that of BDHN-TTF, in agreement with a lower lying highest occupied molecular orbital (HOMO). The HOMO energy levels were experimentally estimated from the first oxidation peak (Table 1) according to: *E*_HOMO_ = –[*E*ox1onset + 4.7] eV (ref. 13 and 14) (Table 1). Also, UV-vis-NIR spectra were recorded for both materials in CH_2_Cl_2_ where the onset of the least energetic band was used to calculate the energy gap between the HOMO and the lowest unoccupied molecular orbital (LUMO).

**Table 1:** Redox potentials (*versus* Ag/AgCl), lowest energy absorption band (*E*_max_) and optical band gap (*E*optg) as well as the experimental and calculated HOMO and LUMO energies (*E*_HOMO_ and *E*_LUMO_) and calculated HOMO–LUMO gaps (*E*calcg) of compounds BDHN-TTF and BN-TTF

Compound		BDHN-TTF	BN-TTF	Δ*E*
Electrochemical data	*E* ox1 1/2 [V]	0.44	0.64	+0.20
	*E* ox2 1/2 [V]	0.92	1.13	+0.21
	*E* ox1 onset [V]	0.33	0.51	+0.18
	*E* exp HOMO [eV]^*a*^	–5.03	–5.21	+0.18
UV-vis data	*E*_max_ [cm^–1^]	21 740	21 410	–320
	*E* opt g [eV]^*b*^	2.70	2.65	–0.05
DFT calculations	*E* calc HOMO [eV]	–4.63	–4.86	+0.23
	*E* calc LUMO [eV]	–1.37	–1.63	+0.26
	*E* calc g [eV]	3.26	3.23	–0.03

^*a*^Estimated from the onset oxidation potential of the first oxidation peak using the empirical equation: *E*_HOMO_ = –[*E*ox1onset + 4.7] eV.

^*b*^Determined from the onset of the lowest energy electronic absorption band in the UV-vis spectrum in CH_2_Cl_2_.

The electronic characteristics of BDHN-TTF and BN-TTF were also calculated by density functional theory (DFT) using the B3LYP functional^15^ and a 6-311G+(d,p) basis set. All reported DFT calculations were carried out with the Gaussian09 code.^16^ As can be seen in Table 1, the experimentally obtained *E*optg and *E*expHOMO values by UV-vis-NIR spectroscopy and CV, respectively, are in reasonable agreement with *E*calcg and *E*calcHOMO. Although the calculated absolute values of *E*_g_ and *E*_HOMO_ tend to be ∼0.4–0.5 eV higher than experimental estimates, the corresponding differences in these energies between the two compounds match very well, in line with our previous studies.^17^

High quality orange block-shaped single crystals of BDHN-TTF were grown by slow evaporation of a solution of the molecule in *p*-xylene. BDHN-TTF crystallized in a solvate-free form in the monoclinic space group *P*2_1_/*c*. The crystal structure contains half of a molecule in the asymmetric unit cell. Each TTF molecule is clearly bended in a chair-like conformation (Fig. 1a). The angle between the planes formed by the TTF core and the external benzene ring is 19.3°. BDHN-TTF crystallises forming layers of molecules in the *bc* plane following a herringbone fashion (*i.e.*, edge-to-face orientation) as can be observed in Fig. 1b. Within these sheets, there are short edge-to-edge-S···S contacts (3.619 Å) along *c*.

**Fig. 1:**
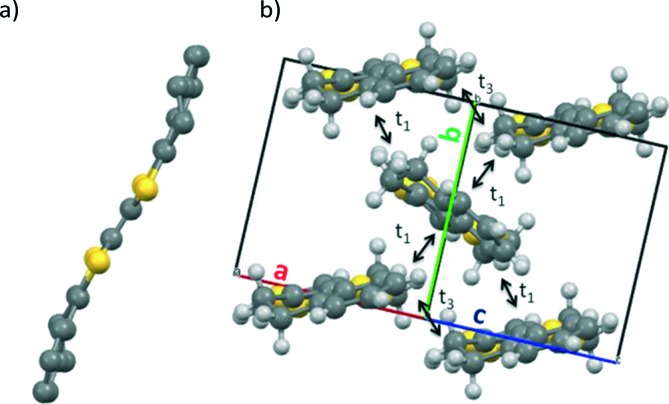
(a) Non-planar BDHN-TTF molecule found in the crystal structure. H atoms are omitted. (b) BDHN-TTF packing following a 2-D herringbone network. The *t*_1_ and *t*_3_ arrows indicate the directions where the transfer integrals have been calculated.

Yellow needle-shaped single crystals of BN-TTF were grown by diffusion of diethyl ether into a solution of the molecule in chlorobenzene. In this case, the molecule crystallizes in the triclinic space group *P*1 and the asymmetric unit cell also consists of half of a BN-TTF molecule, hence, each naphthalene unit is symmetrically equivalent. In the case of BN-TTF, the molecules are very flat, as expected due to its extended aromatic molecular structure (Fig. 2a). The molecules stack in columns along the *a* axis slightly shifted along the long molecular axis, with an interplanar spacing of 3.574 Å (Fig. 2b). Short S···S contacts (3.927 Å) are also found within the stack and between stacks (3.642 Å).

**Fig. 2:**
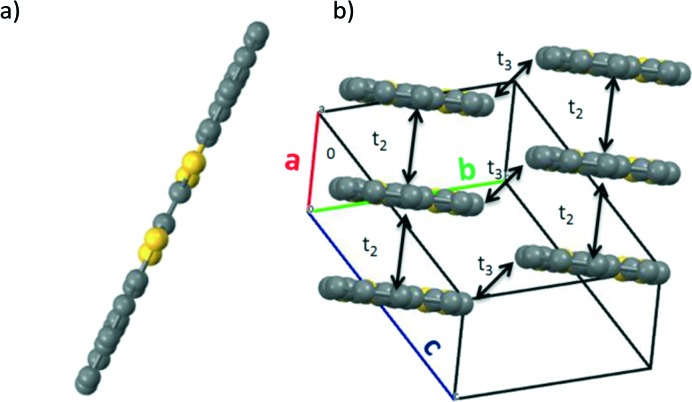
(a) Planar BN-TTF molecule found in the crystal structure. (b) Displaced cofacial packing of BN-TTF showing the calculated electronic couplings (*t*_2_ and *t*_3_). H atoms are omitted for clarity.

Dimers of BDHN-TTF and BN-TTF taken from the resolved crystal structures were used to calculate their hole transfer integrals (*t*). As recommended in ref. 18, DFT calculations using the PW91 functional^19^ were conducted to calculate the HOMO energy splitting of the dimers (*t* for hole conduction is defined as half of the HOMO energy splitting). The calculated values show that a significant electronic interaction is found for the edge-to-face orientation for BDHN-TTF, with a minor edge-to-edge component. In BN-TTF, however, the preferential transport direction is face-to-face, suggesting a strong 1D favoured electronic path (Table 2). Furthermore, the calculated *t* values predict a slightly stronger intermolecular electronic coupling in BDHN-TTF crystals (109 meV) than in BN-TTF (97 meV). On the other hand, the reorganisation energy, calculated at the B3LYP/6-311+G(d,p) level of theory, is only slightly lower in BN-TTF due to its more planar and rigid structure.

**Table 2:** BDHN-TTF and BN-TTF reorganisation energies (*λ*) and most significant HOMO transfer integrals (*t*)

	*λ* (eV)	Edge-to-face, *t*_1_ (meV)	Face-to-face *t*_2_ (meV)	Edge-to-edge *t*_3_ (meV)
BDHN-TTF	0.235	109	—	29
BN-TTF	0.219	—	97	2

OFET devices were fabricated on a bottom-gate bottom-contact configuration using Si/SiO_2_ substrates modified with a self-assembled monolayer of octadecyltrichlorosilane (OTS) with an interdigitated Cr/Au source and drain electrodes (*W* = 25 mm and *L* = 25 μm). The semiconductor was deposited by thermal evaporation (*P* = 9 × 10^–7^ mbar and rate = 0.2–0.3 Å s^–1^).

The evaporated thin films were first characterized by X-ray powder diffraction (XRD) and Atomic Force Microscopy (AFM) (Fig. 3). The diffractogram of BDHN-TTF shows clear equidistant diffraction peaks indicative of the crystallinity of the film as well as the high orientation of the crystals. By comparison with the single crystal structure resolved, it is possible to conclude that both the thin film and the single crystal belong to the same phase. The peaks correspond to the family of *(n00)* reflections and thus, the crystals are oriented with the *bc* plane parallel to the surface, that is, the plane where significant electronic interactions exist (Fig. 3b, left). Accordingly, the AFM images show that the BDHN-TTF films are formed by 2D plate-like structures (Fig. 3a, left).

**Fig. 3:**
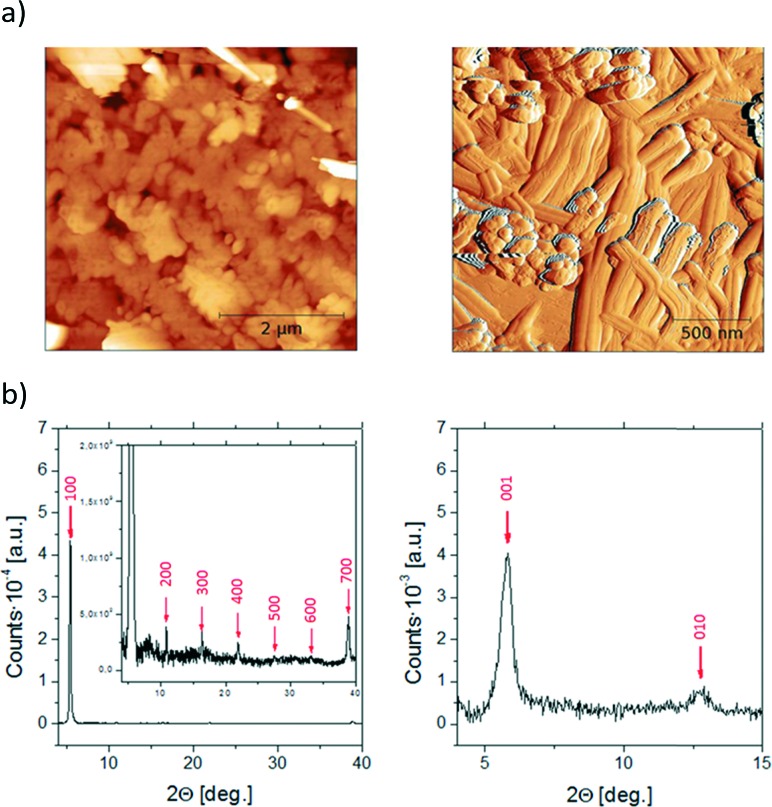
AFM images (a) and X-ray powder diffraction (b) of the evaporated thin films of BDHN-TTF (left) and BN-TTF (right).

The XRD pattern of BN-TTF shows only two peaks that seem to correspond to the (001) and (010) diffractions of the reported single crystal phase (Fig. 3b, right). The AFM images seem to confirm that BN-TTF prefers to form more 1D tape-like structures (Fig. 3a, right). These results point out that the crystals grow with the *a* axis parallel to the surface (*i.e.*, the longest crystal direction of BN-TTF crystals), which corresponds to the stacking direction, which is beneficial for the OFET measurements.

Electrical measurements of the devices were carried out inside a glove box with levels of O_2_ and H_2_O below 1 ppm to avoid the oxidation of the TTFs. Both materials behave as p-type semiconductors, that is, holes are accumulated in the channel by application of a negative gate voltage. BDHN-TTF OFETs exhibit a mobility of 1 × 10^–3^ cm^2^ V^–1^ s^–1^ with a threshold voltage (*V*_TH_) of –11 V and low hysteresis (Fig. 4a). Some contact resistance can be found in the devices probably because of the concentration of defects on the gold–organic semiconductor interface.^20^ BN-TTF OFETs have a mobility of 2 × 10^–3^ cm^2^ V^–1^ s^–1^ with a large positive *V*_TH_ = 33 V after a thermal annealing at 100 °C for one hour (Fig. 4b). Such a large positive threshold voltage has also been typically found for the parent material dibenzo-tetrathiafulvalene (DB-TTF).^21–25^ From the electrical characterisation, it can be concluded that both materials show similar mobility values. We believe that the different electronic dimensionality of the two materials plays a crucial role. The two dimensional character of BDHN-TTF crystals leads to efficient edge-to-face electronic intermolecular interactions and field-effect mobilities comparable to the ones found in BN-TTF.

**Fig. 4:**
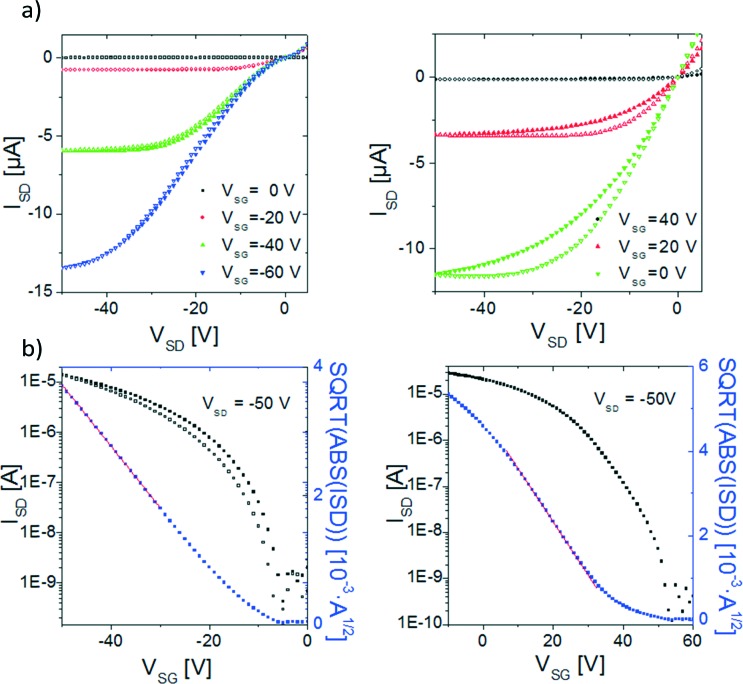
Typical output (a) and transfer (b) characteristics of BDHN-TTF (left) and BN-TTF (right).

## Conclusions

In conclusion, we have successfully characterized the crystal structure of BDHN-TTF and BN-TTF as well as their electronic properties experimentally and theoretically. The reorganization energy values estimated for BN-TTF are reduced with respect to BDHN-TTF revealing that the extension of the π-system should be beneficial for charge transport. However, it is observed that the molecular modifications also have a strong impact on the crystal structure. BDHN-TTF crystallises in a herringbone pattern exhibiting significant intermolecular interactions in the *bc* plane, whilst BN-TTF shows a 1-D cofacial packing. Thin film OFET devices were fabricated with these materials. Both materials exhibited similar device performance which was accounted for by the balance between the calculated reorganisation energy values and the electronic dimensionality. Thus, we demonstrate that molecular engineering is important in order to obtain high mobility materials; however, the crystal structure, which is harder to predict, and, specifically, the electronic dimensionality also play crucial roles.
