# Supplementary material for: Structural and electronic characterisation of π-extended tetrathiafulvalene derivatives as active components in field-effect transistors
Source: CrystEngComm. 2016 Jul 12;18(33):6149–52. doi: 10.1039/c6ce01200k (PMC5059789; doi:10.1039/c6ce01200k)
Supplement: Supplementary file 1 [file CE-018-C6CE01200K-s001.pdf]

Electronic Supplementary Material (ESI) for CrystEngComm.
This journal is © The Royal Society of Chemistry 2016

Supplementary Material (ESI) for CrystEngComm

Supplementary Data for

Structural and electronic characterisation of π-extended tetrathiafulvalene derivatives as active components in field-effect transistors

Antonio Campos,[a] Neil Oxtoby,[a] Sergi Galindo,[a] Raphael Pfattner,[a] Jaume Veciana,[a] Stefan T. Bromley,[b] Concepció Rovira,[a,*] and Marta Mas-Torrent[a,*]

a. Institut de Ciència de Materials de Barcelona (ICMAB-CSIC) and Networking Research Center on Bioengineering, Biomaterials and Nanomedicine (CIBER-BBN), Campus Universitari de Bellaterra, Cerdanyola, E-08193 Barcelona, Spain. Fax: +34 935 805 729; Tel: +34 935 801 853

b. Departament de Química Física & Institut de Química Teòrica i Computacional (IQTCUB) Universitat de Barcelona, E-08028 Barcelona, Spain and Institució Catalana de Recerca i Estudis Avançats (ICREA), E-08010 Barcelona, Spain

*Authors to whom the correspondence should be addressed. E-mail: mmas@icmab.es; cun@ icmab.es;

Table of contents

Experimental details

Synthesis of bis(naphtho[1,2-d])tetrathiafulvalene (BN-TTF).

Figure S1. Cyclic Voltammetry of BDHN-TTF and BN-TTF

Figure S2. UV-Vis spectra

Table S1. Crystal data and refinement details for BDHN-TTF

Table S2. Crystal data and refinement details for BN-TTF

Experimental details

Materials and Methods. Bis(4,5-dihydronaphto[1,2-d])tetrathiafulvalene and other reagents were purchased from Sigma Aldrich and used without further purification. MALDI-TOF MS spectra were recorded on a Bruker Ultraflex II TOF spectrometer. Cyclic voltammetry (CV) was carried out with a traditional three electrode configuration using platinum wires as working and counter-electrode and an Ag/AgCl electrode as reference electrode. These experiments were carried out in a 10^{-4} M solution of BN-TTF or BDHN-TTF in dichloromethane containing 0.1 M of tetrabutylammonium hexafluorophosphate as supporting electrolyte at room temperature. Deoxygenation of the solutions was carried out before the experiments by bubbling Ar during 15 min. UV-vis spectra were carried out in dichloromethane at room temperature at a c = 25 µM using a VARIAN CARY 5000 spectrophotometer The X-ray single-crystal diffraction was measured with a Bruker AXS SMART APEX using graphite-monochromated Mo Kα radiation (λ = 0.71073 Å). The measurements were performed with θ in the range 2.52°-28.28° for BDHN-TTF and 2.61°-28.25° for BN-TTF. Full-sphere data collection was carried out with f and w scans. Programs used: data collection, Smart version 5.631 (Bruker AXS 1997-02); data reduction, Saint + version 6.36A (Bruker AXS 2001); absorption correction, SADABS version 2.10 (Bruker AXS 2001). Structure solution and refinement was done by using SHELXTL Version 6.14 (Bruker AXS 2000-2003). The structure was solved by direct methods and refined by full-matrix least-squares methods on F2 non-hydrogen atoms were refined anisotropically. The H-atoms were placed in geometrically optimized positions and forced to ride on the atom to which they are attached. Substrates for thin film OFETS were prepared by photolithography using a Micro-writer from Durham Magnetico Optics LTD and afterwards a evaporation of Cr (5 nm) and Au (40 nm) with a Evaporation System Auto 306 from Boc Edwards at $P=2\cdot10^{-6}$ mbar. Thin film OFETs were fabricated on a bottom-gate bottom-contact configuration using Si substrates with 200 nm thermally grown SiO_2. The substrates were modified with a self-assembled monolayer of octadecyltrichlorosilane (OTS). Interdigitated source and drain electrodes (W = 25 mm and L = 25 µm) were fabricated by photolithography using a Micro-writer from Durham Magnetico Optics LTD and afterwards by evaporation of Cr (5 nm) and Au (40 nm) with a Evaporation System Auto 306 from Boc Edwards at $P=2\cdot10^{-6}$ mbar. The semiconductor was deposited by thermal evaporation ($P = 9\cdot10^{-7}$ mbar and rate= 0.2-0.3 Å/s) using an evaporator UNIVEX 350G from Oerlikon. Film thickness' were monitored during the evaporation by the quartz sensor inside the chamber and for both cases it was 50 nm. X-ray powder diffraction analysis was carried out in a PANalytical X'PERT PRO diffractometer MRD. AFM images were obtained using

a 5100 SPM system from Agilent technologies. Electrical characterisations of the films were carried out inside a glovebox under inert conditions (O_2 and H_2O below 1 ppm) using a Keithley 2612A Source Meter.

Synthesis of bis(naphtho[1,2-d])tetrathiafulvalene (BN-TTF). Bis(4,5-dihydronaphto[1,2-d])tetrathiafulvalene (615 mg, 1.5 mmol) and DDQ (516 mg, 2.3 mmol) were placed in dry glassware and flushed with $Ar_{(g)}$. Freshly dried toluene (50 mL) was added and the solution was left to reflux under an inert atmosphere for 20 hours. Afterwards, the solution was cooled to room temperature and filtered. The resulting solid was washed with THF and purified by recrystallisation in dry toluene giving BN-TTF in a 61% yield. m.p. 284-285 ^{o}C; IR (ATR-IR) $\bar{\upsilon} =$ 3050, 2955, 2920, 2840, 1737, 1618, 1578, 1557, 1499, 1376, 1334, 1256, 1203, 1116 cm^{-1}; ^{1}H NMR (600 MHz, CD_2Cl_2, R.T.): δ = 7,88 (d, 4H), 7,57-7.67 (m, 4H), 7.41-7.52 (m, 4H); ^{13}C NMR data are unavailable due to its poor solubility; MS (MALDI-TOF) (m/z) calculated for $C_{22}H_{12}S_4$: 403.98 found: 404.09.

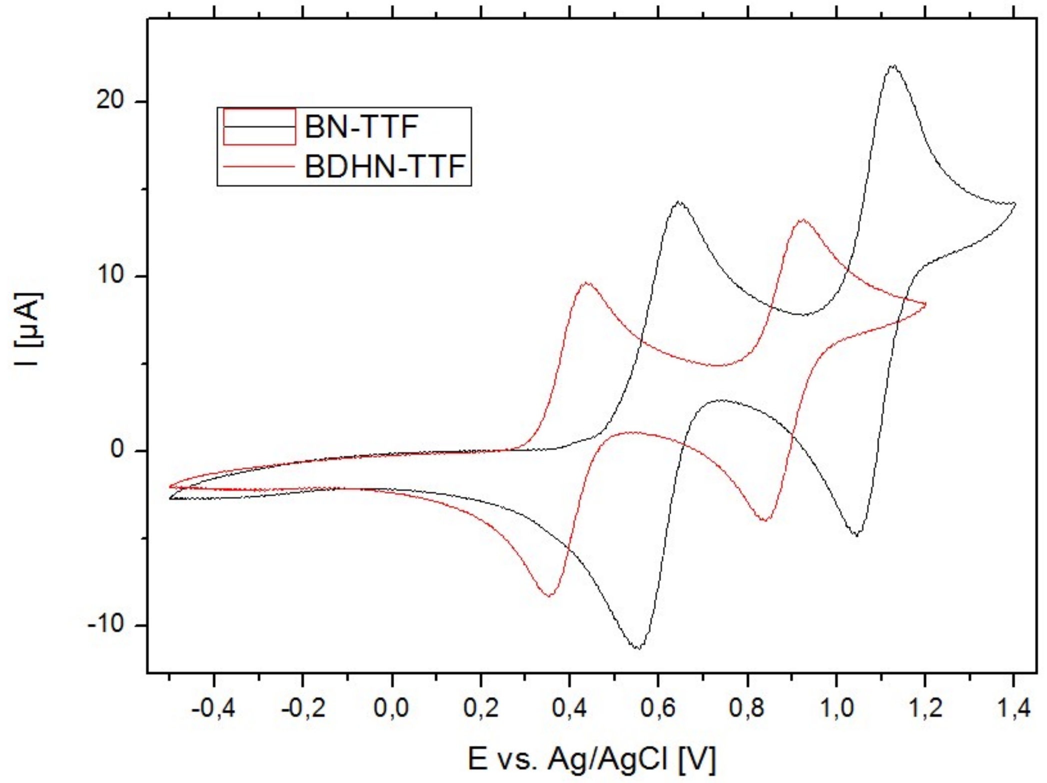

Figure S1. Cyclic voltammetry of BN-TTF and BDHN-TTF.

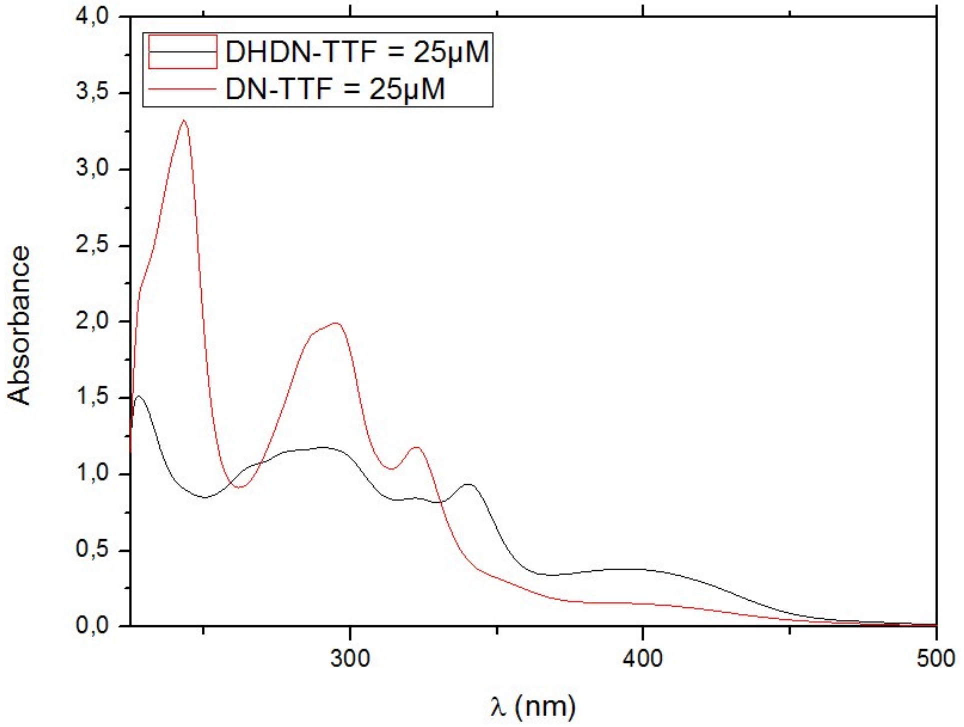

Figure S2. UV-vis spectra of BN-TTF and BDHN-TTF.

Crystal system	Monoclinic
Space group	P21/c
Formula	$C_{22}H_{16}S_4$
Cell length a	16.1748(18)
Cell length b	7.7639(9)
Cell length c	7.5390(8)
Cell angle alpha	90
Cell angle beta	91.191(2)
Cell angle gamma	90
Cell volume	946.54(18)
Cell formula units Z	2
Cell measurement temperature	300(2)
Crystal description	block
Crystal colour	orange
Crystal size max	0.08
Crystal size mid	0.06
Crystal size min	0.02
R	0.0360
wR	0.1062

Table S1. Crystal data and refinement details for BDHN-TTF.

Crystal system	Triclinic
Space group	P-1
Formula	$C_{22}H_{12}S_4$
Cell length a	13.9274(14)
Cell length b	7.167(3)
Cell length c	15.716(6)
Cell angle alpha	83.267(6)
Cell angle beta	89.379(6)
Cell angle gamma	80.859(6)
Cell volume	433.7(3)
Cell formula units Z	1
Cell measurement temperature	300(2)
Crystal description	Needle
Crystal colour	Yellow
Crystal size max	0.1
Crystal size mid	0.05
Crystal size min	0.05
R	0.0492
wR	0.1131

Table S2. Crystal data and refinement details for BN-TTF.
